# Bacillary Positive Tuberculous Body Fluid Smears: A Perspective on How Fast to Use Acid Fast

**DOI:** 10.7759/cureus.38447

**Published:** 2023-05-02

**Authors:** Bidish K Patel, Debasis Gochhait, Karthik Dhandapani, Temjen Sunup Jamir, Praveena Edura, Divya Parepalli, Neelaiah Siddaraju

**Affiliations:** 1 Pathology, Jawaharlal Institute of Postgraduate Medical Education and Research, Puducherry, IND; 2 Research, Massachusetts General Hospital Cancer Center, Boston, USA; 3 Oncopathology, Gujarat Cancer Research Institute, Ahmedabad, IND; 4 Pathology, North Eastern Indira Gandhi Regional Institute of Health and Medical Sciences, Shillong, IND

**Keywords:** transudative effusion, exudative effusion, caseous necrosis, bacillary positive effusion, tuberculous ascites, tuberculous pleural effusion, acid fast, bacilli positive, fluid cytology, tuberculous effusion

## Abstract

Background

Tuberculous effusions are common. Classically, they are described as bacteria poor and lymphocyte rich. Our experience, however, has been more varied. We compiled this rare group of bacteria-positive tuberculous fluids to document their cytologic spectrum and to look for possible predictors of bacillary positivity.

Methods

Fifty-one cases of bacillary positive fluids were identified and their clinicopathological details were noted. Per case, the smear background was assigned as either clear, caseous, suppurative, granular proteinaceous or frankly hemorrhagic. Fine, punched-out vacuoles in the smear background were also noted. The bacillary load in each case was classified from scanty to 3+. Eventually, the clinicopathologic variables were tabulated for frequency and studied for any association with bacillary presence.

Results

Only 19 of the 51 patients had a history of tuberculosis. Retropositive patients comprised a small proportion (9.8%) and did not always indicate strong (3+) bacillary positivity. The granular proteinaceous background was the most frequent (35%) pattern. Only a suppurative background was associated with strong bacillary positivity. Fine vacuoles were seen almost always with caseous and granular proteinaceous backgrounds but without statistical significance.

Conclusion

Tuberculous effusions can have diverse smear backgrounds, not necessarily one rich in caseous material. When tuberculosis is known or clinically suspected, non-classical findings such as abundant neutrophils or suppurative background should not dissuade one from requisitioning mycobacterial stains. In fact, acid-fast stains should probably routinely accompany Giemsa slides of clinically idiopathic effusions in endemic areas since it is still the cheapest and fastest method for a conclusive diagnosis.

## Introduction

Tuberculosis, with the increased prevalence of immunosuppressed states, has made a comeback even in developed countries. Despite coordinated global efforts to eradicate tuberculosis, it still constitutes a major health problem. In 2021, its incidence was an estimated 10.6 million cases (0.7 million in people living with HIV/AIDS) and it contributed to 1.4 million deaths worldwide. This is the thirteenth most prolific cause of mortality across all nations. The prevalence varies across regions and countries. In Asia, for example, the prevalence varies from a low of 119 (in China) to a high of 1159 per 100,000 population in the Philippines [1]. Among the extrapulmonary manifestations, involvement of lymph nodes and effusions are the most common [2].

Demonstration of acid-fast bacilli (AFB) in tissue specimens is the gold standard in the diagnosis of tuberculous etiology. Unfortunately, attempting the same in body fluids can be quite a challenge. This is because, for a long time tuberculous effusions have been attributed to only a hypersensitivity reaction against the bacillary proteins, a theory which in turn is supported by the near lack of demonstrable bacilli in the body fluids (except sometimes in empyema or immunosuppressed states as HIV) [2-7]. In fact, the overall sensitivity of pleural fluid smears in detecting acid-fast bacilli is stated to be a measly less than 10% [7].

Of late, the abovementioned conventional thinking has been put to the test by evidence to the contrary in the form of recent advances in laboratory diagnosis. With the increasing availability of techniques such as liquid culture, polymerase chain reaction (PCR) and NAAT (nucleic acid amplification testing) the sensitivity of detecting the organism in fluids has increased [2]. Yet, given the cost and accessibility of these ancillary techniques, demonstrating real bacilli on fluid cytology should still be treated as a priority and a possibility not to be taken lightly.

Against this background, we retrospectively reviewed our cases of proven AFB-positive tuberculous body fluids to prepare a clinicopathological narrative and to ultimately look for subtle morphological clues that would help a practising cytopathologist judiciously predict bacillary positivity.

## Materials and methods

For this retrospective study, fluid cytology reports of the past ten years (January 2007 to December 2016) were reviewed. Sixty-four such cases were found over a period from January 2007 to December 2016. Subsequently, the requisition forms and stained slides were retrieved from our departmental archives. Cases without either were excluded from further study. Eventually, 51 cases with complete archival material were included in the study. The age, gender, retropositive status at presentation, known prior/current tuberculous infection and presence of pneumothorax (if any) were the key points noted from history. No new stain/patient intervention was performed for the study and patient’s identities are not disclosed. For such studies, institute review board permission is waived in our institute.

At our institute, every case submitted for cytological evaluation of body fluids undergoes staining for Papanicolau stain and May-Grünwald Giemsa (MGG) stains. Subsequently, depending on cytomorphological clues or clinical suspicion, Ziehl-Neelsen (ZN) stain for acid-fast bacilli and PAS (Periodic acid Schiff) stain for atypical mycobacteria/fungal elements are performed. To elaborate, the samples that undergo compulsory screening with mycobacterial stains include known patients of tuberculosis, retropositive patients (patients that have had a positive antigen test for HIV-1 or HIV-2 virus), patients on chronic immunosuppressant therapy for any reason, turbid effusions or samples comprising frank pus. On microscopy, idiopathic lymphocyte rich effusion having many reactive lymphocytes (larger than mature lymphocytes (1.5-2X) with prominent bluish cytoplasm and paler nuclear chromatin) are also screened for tuberculous bacilli. All stains used are conventional versions prepared and stained as per standard protocols.

The *gross* nature of the fluids is assessed by cytotechnologists and noted on the form. These were noted, suitably condensed and coded (on a Microsoft Office Excel Worksheet, version 2007, Redmond, WA, USA) as clear/straw coloured, turbid/suppurative, frank pus and hemorrhagic.

The focus of the *microscopic* evaluation was the MGG smear background with lesser emphasis on cellular constituents. Post-assessment, the cases were categorized into five groups. These were:

Pattern 1: *Clear* (thin proteinaceous background with minimal, if any, RBCs)

**Figure 1:**
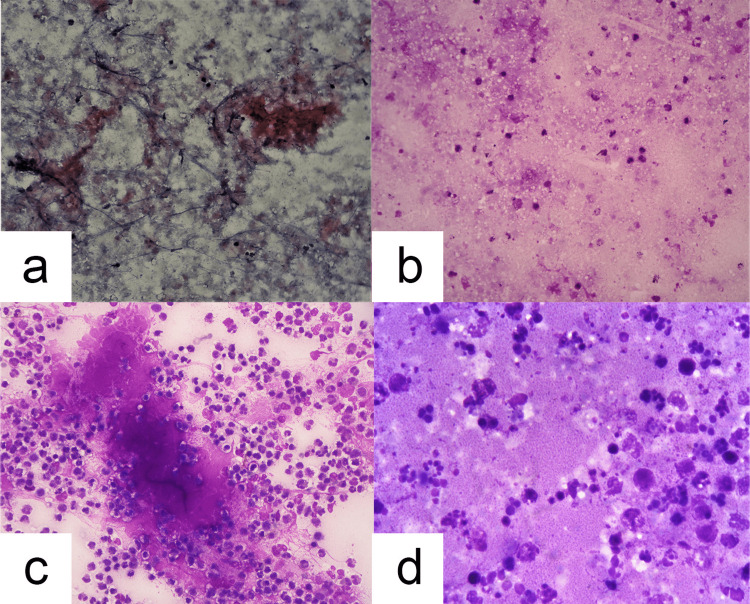
Varied smear backgrounds in tuberculous body fluids. (a) Frank caseous material (Papanicolau stain, 100X). (b) Thin caseous background.  Fine, punched-out negatively stained vacuoles are seen, especially prominently in the left upper quadrant (May Grünwald Giemsa stain, 100X). (c) Suppurative background with predominantly intact neutrophils, thin streaks of scattered pus and a small patch of caseous-like material (May Grünwald Giemsa stain, 100X). (d) Granular proteinaceous background with abundant karyorrhectic debris and only a few, if any, intact neutrophils (May Grünwald Giemsa stain, 1000X).

Pattern 2: *Caseous* (frankly fine, powdery azure positive material and generally cell poor) (Figure 1a, 1b)

Pattern 3: *Suppurative* (stringy, necrosed material with abundant intact neutrophils) (Figure 1c)

Pattern 4: *Granular proteinaceous* (coarse granular debris seen all over a proteinaceous magenta background. Smear is generally rich in karyorrhectic neutrophils) (Figure 1d)

Pattern 5: *Frankly hemorrhagic* (comprising overwhelmingly of blood elements with insignificant patches of necrotic material)

Of note, there was often an overlap of these categories. In these situations, the predominant category (defined arbitrarily as the one that is present in roughly >80% of the smear area) was accorded to the case. In the smears with a clear background, we noted the predominant cell in the slide.

A summary of these abovementioned patterns is provided in Table 1.

**Table 1:** The five varied smear background patterns observed in bacillary positive tuberculous effusion smears.

Background pattern	Cellular component		Non-cellular component	
	Cellularity	Predominant cellular component	Background	Other clues
Clear (Pattern 1)	low/medium	lymphocytes	thin, proteinaceous (pale, magenta colored)	-homogeneous texture
Caseous (Pattern 2)	very low/low	lymphocytes	amorphous, powdery to clumpy (variably dense magenta colored)	-acellular -pinhead or smaller-sized, clear circular spaces resembling vacuoles frequently seen. These impart a bubbly/foamy appearance to the background.
Suppurative (Pattern 3)	high	intact neutrophils	streaky and thready, dense, dark magenta-colored pus	-neutrophils entrapped in pus clumps
Granular proteinaceous (Pattern 4)	high	fragmented neutrophils	granular, spotty, deep magenta-colored background	-rich in karyorrhectic nuclear debris
Hemorrhagic (Pattern 5)	low	degenerating/intact red blood cells	thin, proteinaceous plasma (magenta colored)	-reminiscent of a blood smear -cells often show artefactual changes (vacuolar degeneration - white cells; fragmentation and water artefact - red cells)

Another feature we intended to focus on, based on our past observations, was the presence of fine, punched-out vacuoles in the background giving a foamy/bubbly appearance (Figure 1b). We noted them if they were obvious to the aided eye in an MGG smear in at least three out of 10 consecutive high-power fields for 20 such fields in two different areas.

Without any prior established system of grading for acid-fast bacilli on body fluids and given the general paucity of bacilli on fluid smears, we decided to implement our own arbitrary modification of the Ridley’s logarithmic scale for bacteriological index assessment of lepra bacilli to classify the strength of bacillary positivity. This was preferred over the system used to grade (the usually more copious) tubercle bacilli in sputum smears [8,9].

In our proposed system, the positivity was graded by counting acid-fast bacilli (as seen on a ZN-stained smear) over 100 consecutive oil immersion fields and averaging them, as follows:

Scanty = Only one bacillus in 100 fields

Grade 1+ = 2-10 bacilli per 100 fields

Grade 2+ = 11-100 bacilli per 100 fields

Grade 3+ = more than 100 bacilli per 100 fields

Grade 3+ cases were taken as *strong* positivity.

Tabulation and calculation of averages were done using Microsoft Office Excel Worksheet (version 2007, Redmond, WA, USA). The statistical association was calculated using the two-tailed Fisher’s exact test on the GraphPad QuickCalcs webpage (http://www.graphpad.com/quickcalcs/contingency2/, accessed on June 17, 2017). A p-value of <0.05 was considered to be statistically significant.

## Results

**Table 2:** Cytopathological and demographic features in relation to five different smear backgrounds observed in bacillary positive tuberculous body fluids

Demographic and cytopathological parameters		Types of background patterns					
		Clear (Pattern 1)	Caseous (Pattern 2)	Suppurative (Pattern 3)	Granular Proteinaceous (Pattern 4)	Frankly hemorrhagic (Pattern 5)	
% of cases (no. of cases)		20(10)	16(8)	25(13)	35(18)	4(2)	
Mean age (rounded off to the nearest year)		35	36	42	42	38	
Age range (in years)		18-60	22-47	10-65	25-67	35-40	
Male:Female		9:1	7:1	5.5:1	8:1	1:1	
Type of Fluid [% (no. of cases)]	pleural	70(7)	61(5)	77(10)	78(14)	100(2)	
	peritoneal	20(2)	13(1)	-	11(2)	-	
	pericardial	10(1)	13(1)	-	-	-	
	synovial	-	13(1)	23(3)	11(2)	-	
Gross appearance [% (no. of cases)]	clear	70(7)	-	-	-	-	
	turbid	30(3)	50(4)	23(3)	100(18)	-	
	pus	-	50(4)	77(10)	-	-	
	hemorrhagic	-	-	-	-	100(2)	
Vacuoles in background [% (no. of cases)]		-	88(7)	8(1)	72(13)	-	
Retropositive patients (number of cases)		2	1	1	1	-	
Strength* of positivity for AFB [% (no. of cases)]	Scanty	60(6)	63(5)	31(4)	67(12)	100(2)	
	1+	30(3)	25(2)	15(2)	16(3)	-	
	2+	-	-	15(2)	11(2)	-	
	3+	10(1)	12(1)	39(5)	6(1)	-	

Over a decade, a total of 51 cases were identified that had complete archival material and available requisition forms. Nineteen of these (37%) were known cases of tuberculosis, either under active treatment or with a known history. Only a bland “effusion for evaluation” without differentials comprised 12 (24%) patients while the rest of the 20 (39%) patients had suggested differentials including infective etiology, malignant effusion, autoimmune diseases and probable hematolymphoid malignancy. Five patients were known retropositives (patients that have had a positive antigen test for HIV-1 or HIV-2 virus) on antiretroviral therapy. About a quarter (12 cases; 24%) of the patients had evidence of pneumothorax along with evidence of effusion.

A majority of the fluids received for evaluation were from a pleural effusion (38 cases; 75%). Pericardial, peritoneal and synovial fluids comprised two, five, and six cases, respectively.

Males predominated the cohort, comprising 86% (44 patients) of all cases. The average age of patients studied in the series was 40 years. Women (average - 33 years) were affected at an overall younger age than men (average - 41 years). The youngest and the oldest patients were both males, aged 10 and 67 years, respectively.

The various demographic and important pathological parameters in correlation with the various background patterns are summarized in Table 2.

**Table 3:** Cytopathological and demographic features in relation to five different smear backgrounds observed in bacillary positive tuberculous body fluids. *Positivity was graded based on acid-fast bacilli (AFB) counted over 100 consecutive oil immersion fields as follows: Scanty = 1 bacilli; Grade 1+ = 2-10 bacilli; Grade 2+ = 11-100 bacilli and Grade 3+ = more than 100 bacilli per 100 fields.

Demographic and cytopathological parameters		Types of background patterns				
		Clear (Pattern 1)	Caseous (Pattern 2)	Suppurative (Pattern 3)	Granular Proteinaceous (Pattern 4)	Frankly hemorrhagic (Pattern 5)
% of cases (no. of cases)		20(10)	16(8)	25(13)	35(18)	4(2)
Mean age (rounded off to the nearest year)		35	36	42	42	38
Age range (in years)		18-60	22-47	10-65	25-67	35-40
Male:Female		9:1	7:1	5.5:1	8:1	1:1
Type of Fluid [% (no. of cases)]	pleural	70(7)	61(5)	77(10)	78(14)	100(2)
	peritoneal	20(2)	13(1)	-	11(2)	-
	pericardial	10(1)	13(1)	-	-	-
	synovial	-	13(1)	23(3)	11(2)	-
Gross appearance [% (no. of cases)]	clear	70(7)	-	-	-	-
	turbid	30(3)	50(4)	23(3)	100(18)	-
	pus	-	50(4)	77(10)	-	-
	hemorrhagic	-	-	-	-	100(2)
Vacuoles in background [% (no. of cases)]		-	88(7)	8(1)	72(13)	-
Retropositive patients (number of cases)		2	1	1	1	-
Strength* of positivity for AFB [% (no. of cases)]	Scanty	60(6)	63(5)	31(4)	67(12)	100(2)
	1+	30(3)	25(2)	15(2)	16(3)	-
	2+	-	-	15(2)	11(2)	-
	3+	10(1)	12(1)	39(5)	6(1)	-

In terms of frequency, the overall order of smear background patterns seen was Pattern 4 > Pattern 3 > Pattern 1 > Pattern 2 > Pattern 5. Thus, pattern 4 (granular, proteinaceous background) was the commonest contributing slightly over a third of all cases (35%). Patterns 3 and 4 were seen in slightly older patients. None of the ascitic fluid samples showed patterns 3 and 5, while none of the pericardial fluid samples had patterns 3 and 4. Synovial smears never showed clear background (pattern 1). Gross examination showed some minor discordance in the initial three categories.

The presence of minute vacuoles in the background (giving a bubbly/foamy appearance) was seen in a total of 21 patients (41%), a large majority in patterns 2 and 4. One each of the ascitic and pericardial fluids and two synovial fluids sampled showed these vacuoles. The rest 17 samples (33%) were of pleural fluid.

**Figure 2:**
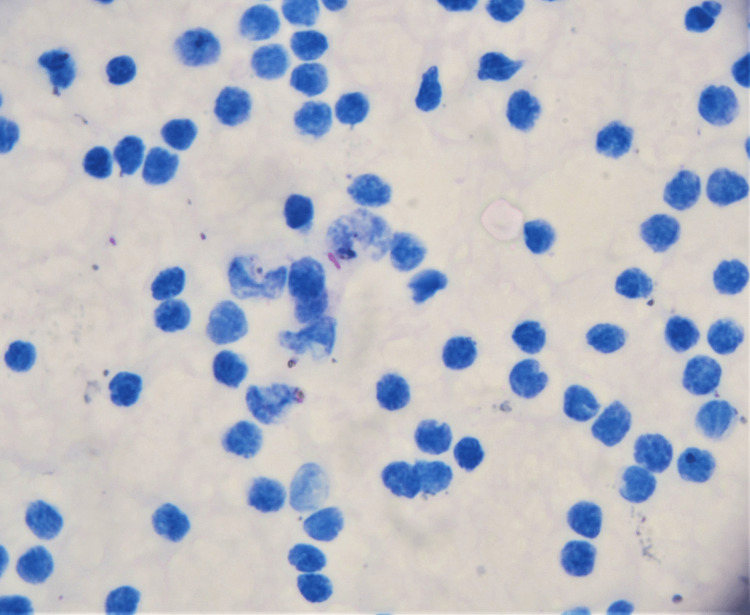
Bacillary positive transudative effusion. A rare rose-pink acid-fast bacillus seen here in the middle of the field in a tuberculous ascites sample (Ziehl-Neelsen stain, 1000x).

The bacillary load was predominantly scanty (29 cases; 57%) (Figure 2). Grade 1 and 2 positivity was seen in 10 cases (19%) and four cases (8%), respectively.

**Figure 3:**
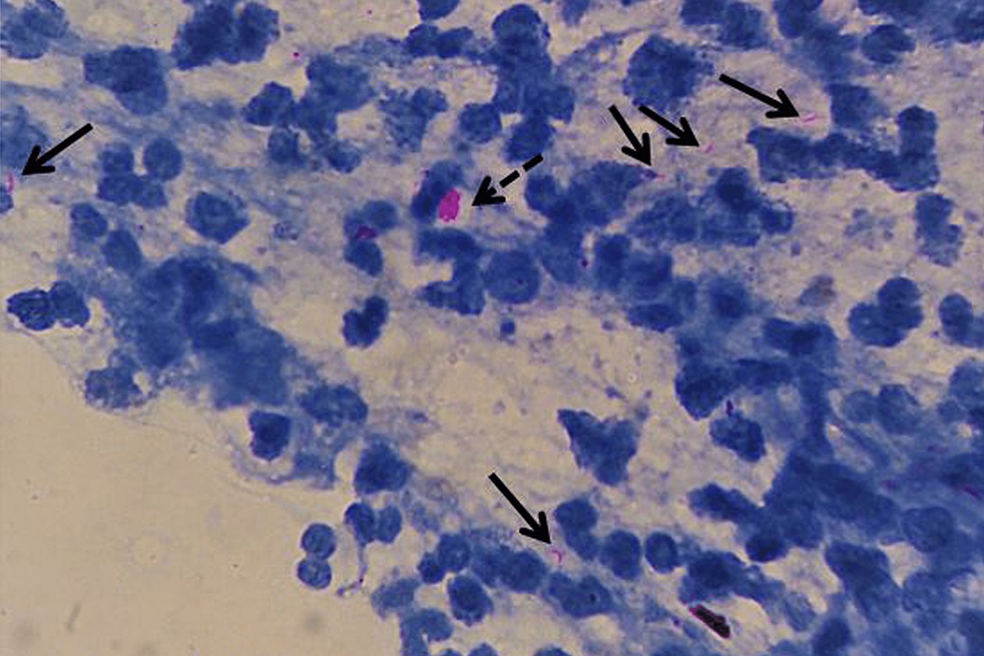
Strong acid-fast bacillary positivity in an effusion smear with a suppurative background. Strong positivity demonstrated as multiple intact and fragmented acid-fast bacilli (solid black arrows) in the smear as well as a clump of tubercular bacilli (dashed black arrow), resembling a globi of lepra bacilli (Ziehl-Neelsen stain, 1000X).

Of those demonstrating strong bacillary positivity (eight cases; 16%), all were males and a majority comprised patients with pattern 3, i.e., a suppurative pattern (Figure 3). In fact, this association was statistically significant (p = 0.019). Also, the bacillary load was mostly scanty in all other patterns, including smears rich in caseous necrotic material. Contrary to expectations, punched-out vacuoles in the smear background were seen in only two of the eight strongly positive cases. With a p-value of 0.14, this finding was thus not associated with strong positivity. A summary of patients with strong positivity for AFB is provided in Table 3.

**Table 4:** Clinicopathological features in the cases of tuberculous body fluids which showed strong* (3+) bacillary positivity. * More than 100 bacilli per 100 consecutive oil immersion fields seen in a Ziehl-Neelsen stained smear.

Serial Number	Age (in years)	Type of fluid	Smear background	Bubbly background	Retropositive status
1	10	Synovial	Suppurative	Not seen	No
2	23	Pleural	Suppurative	Not seen	No
3	34	Pericardial	Clear	Not seen	Yes
4	35	Pleural	Caseous	Seen	Yes
5	38	Pleural	Granular proteinaceous	Not seen	No
6	45	Pleural	Suppurative	Not seen	No
7	48	Pleural	Suppurative	Not seen	No
8	60	Pleural	Suppurative	Seen	Yes

The salient features with regard to retropositive patients are presented in Table 4. Five patients (10%) were known to be retropositive in our study at the time of presentation. All these patients were males. Four of them were in the fourth decade of life. Two of the five patients did not exhibit strong bacillary positivity. In fact, both had only a 1+ bacillary positivity. Only those with pleural effusion demonstrated a bubbly background rich in tiny vacuolar spaces. No case showed atypical mycobacteria, i.e., PAS-positive bacilli.

**Table 5:** Clinicopathological features in the retropositive cases with bacillary positive tuberculous body fluids. *Positivity was graded based on acid-fast bacilli (AFB) counted over 100 consecutive oil immersion fields as follows: Grade 1+ = 2-10 bacilli; Grade 2+ = 11-100 bacilli and Grade 3+ = more than 100 bacilli per 100 fields.

Serial Number	Age (in years)	Type of fluid	Smear background	Bubbly background	AFB positivity*
1	31	Pleural	Granular proteinaceous	Seen	1+
2	34	Pericardial	Clear	Not seen	3+
3	35	Peritoneal	Clear	Not seen	1+
4	35	Pleural	Caseous	Seen	3+
5	60	Pleural	Suppurative	Seen	3+

## Discussion

Tuberculous effusions are more a norm than an exception in chest medicine clinics of underdeveloped /developing countries. Despite the decreasing trends in incidence, with HIV infection, increasing globalization and travel and increasing use of immunosuppressants (steroids, drugs for autoimmune diseases and drugs to prevent transplant rejection), tuberculosis is unlikely to be eradicated anytime soon [10]. It is imperative, therefore, to be aware of when to clinically as well as cytologically suspect tuberculous etiology in effusions.

Demonstrating the organism is the gold standard for diagnosis but direct smears have a very low sensitivity [7]. Conventional wisdom thus dictates resorting to pleural biopsy or culture or more sophisticated techniques such as nucleic acid testing or PCR for diagnosis because of their higher diagnostic yield. But they are not without their own share of problems. A pleural biopsy may be non-representative or inadequate in untrained hands along with the attendant surgical complications; cultures take weeks while the advanced tests cost money and are not yet widely available in many laboratories [2,3,11-13]. Given the fact that the population most susceptible to tuberculosis is the one that’s the furthest from such high-end care, a simple smear examination and diligent screening of acid-fast bacilli in a basic pathology laboratory may sometimes make all the difference.

To the best of our knowledge, there has not been any large study focusing on cytological aspects of bacilli-positive tuberculous effusions. Documentation of bacilli positivity on direct smears of tuberculous fluids is few and far between. The range of positivity we came across in our literature search was <10% in pleural, ≤42% in pericardial, ≤3% in peritoneal, and up to 5% in synovial fluids [7,14-19]. This paucity of data and the rare nature of bacillary positivity prompted us to plan a study that would help in the prudent ordering of mycobacterial stains and in signing out an early conclusive report.

A large majority of our patients were males (87%) in the fourth and fifth decades. A quarter of the patients had evidence of pneumothorax apart from pus/effusion. This has not been mentioned as a prominent feature in our literature search and may be an illustration of the late and complicated presentation common in countries with low socioeconomic status.

Generally, a frankly hemorrhagic or even serosanguinous fluid is unlikely to be tuberculous [20,21]. Our study had only two such cases. Up to 42 of 51 fluids in our study were turbid (implying necrosis) to frankly necrotic to the naked eye. The gross nature of fluids is noted by our technologists on receipt of fluid specimens. This may account for some discordance between micro- and macroscopic findings. For example, three samples with microscopically clear background had appeared turbid on reception. All these smears did possess blotches of RBCs and few karyorrhectic neutrophils in them, which may explain the macroscopy. In retrospect, they were probably serosanguinous. Of note, only 14% (seven cases) of our fluids at presentation were clear, unlike more than 80% as quoted in the literature [13].

At microscopy, available literature goes by the norm that pleural fluid lymphocytosis (often >80%), a near lack of mesothelial cells and paucity of eosinophils (<10% of cells) are almost the *sine qua non* for diagnosis [22-24]. One must understand that these values are based on transudative effusions where the organisms were diagnosed mainly by culture positivity, pleural histopathology and other such sensitive methods. On the other hand, our cases include all “fluid samples” with bacillary positivity irrespective of whether they were transudates (clear fluids) or exudates (empyema). Thus, our cases mostly represent an extreme of pathophysiology where the disease is at an advanced, neglected stage manifesting with such complications as emphysema and hydro-/pyopneumothorax. This would explain the preponderance of turbid and pus-like samples in our series. Logically, in such cases the bacillary load is likely to be high and less sensitive methods such as smear AFB will suffice. This hypothesis would concur with studies that have proven polymorphonuclear cell-rich tuberculous effusions have more chances of microbiological and sputum bacillary positivity as well a poorer prognosis [22,25,26].

With that knowledge, our focus turned from counting cellular constituents to the smear background, which is less cumbersome and more reproducible. This is because procedure-related hemorrhage with subsequent cellular contamination, autolysis from delayed processing and potential for confusion among cellular constituents such as macrophages, mesothelial cells and reactive lymphocytes do not influence smear background. With an arbitrary criterion of 80% of smear area to assign the "predominant pattern", an element of objectivity was introduced that took care of overlapping categories. As stated earlier, the background was then categorized into five patterns. As expected, the majority of the patients had evidence of suppurative necrosis indicative of an empyema [7]. Naturally, it equated with neutrophils being the predominant cells in the background.

A special mention of pattern 1 (clear background) pleural fluids is warranted since they represent the form of effusion laboratories of the western hemisphere are perhaps more used to. No less than a fifth of our cases (10 cases) were in that category. Thus, a crucial take-home message would be to realize that AFB positivity is possible in transudative effusions (Figure 2). The predominant cellular elements in these smears were reactive lymphocytes (three cases), mature lymphocytes (four cases), neutrophils (two cases) and reactive mesothelial cells (one case). At times, reactive lymphocytes and an occasional plasma cell were seen in fluids with a clear, proteinaceous background.

Tuberculous body fluids, especially pleural effusion generally do *not* show plenty of mesothelial cells [27]. The reason is attributed to the formation of fibrinopurulent exudate coating the pleural cavity and covering the mesothelium. We observed a prominent mesothelial population in only one case (2%). Koss mentions in his book a polar opposite situation with florid mesothelial hyperplasia giving rise to the suspicion of mesothelioma or metastasis [28]. Our lone case showed reactive changes with nucleolus and abundant cytoplasm, but they did not present a diagnostic dilemma.

Over the years, fine and punched-out, unstained small vacuoles in the smear background had been observed by one of our authors in tuberculous lesions from many sites (Figure 1b). The probable origin may be related to mycolic acids (a fatty acid) on the mycobacterial cell wall. The corollary or utility being more the bacillary load more would be the lipoid vacuoles shed from dying bacilli. In our study, we observed them to be common in patterns 2 and 4 where the necrotic material had a granular texture. However, the vacuoles did not correlate with a strong positivity of AFB (p = 0.14) as we had hoped for. Further studies may shed more light on these enigmatic findings.

With our observations, we can now recommend that fluids which are frank pus grossly (representative of a microscopic pattern 3) need to have an upfront ZN-stained slide submitted with the MGG-stained smear. There is a statistically significant association of strong positivity (p = 0.014) as well as at least a 1+ positivity (p = 0.049) in cases with pattern 3. Among the rest, clinical history of old, untreated/partially or completely treated TB or a strong clinical suspicion should also require ZN staining straightaway.

While beyond the scope of our study, we hope future studies attempt bacilli demonstration after concentration methods/cell block preparation of the sediment and/or fluorescent stains. Any of these methods, if fruitful, will certainly improve bacillary detection in body fluids. It is important to note that only conventional direct smears should be employed for ZN staining since liquid-based cytology (LBC) reagents are known to lyse acid-fast bacilli [29,30].

The study group being a retrospective bacillary positive cohort represents a limitation of this study. Designing a case-control study including bacillary negative tuberculous effusions would have allowed evaluation of the strength of association of the various types of background with bacillary positivity. However, a lack of concrete numbers of the false negative smear cases because of poor cataloguing of microbiological data and only the recent availability of PCR testing/NAAT (Nucleic Acid Amplification Test) at our center discouraged us from such an attempt.

## Conclusions

To summarize, any pleural tap yielding pus/pus like turbid fluid does not necessarily equate to a conventional (coccoid) bacterial etiology, but instead may represent tuberculous empyema. We could demonstrate five patterns of smear background in bacillary positive body fluids of which the ones with suppurative background are likely to yield stronger bacillary positivity. Even transudative effusions may harbor bacilli. Tiny, punched out vacuoles in the background were frequently seen in our cases but their current utility is questionable.

Our study is designed to be of practical utility to especially those cytopathologists who work in small laboratories with technological constraints and have access to at best, rudimentary clinical information. We hope this work makes readers view smear backgrounds in a new light in the context of a tuberculous etiology. Combined with a high index of morphological suspicion, a fast resort to mycobacterial stains is worthwhile since they still remain the most time- and cost-effective method in establishing the diagnosis.
